# Supplementary material for: WordCommentsAnalyzer: A windows software tool for qualitative research
Source: F1000Res. 2018 Sep 4;7:536. Originally published 2018 May 3. [Version 2] doi: 10.12688/f1000research.14819.2 (PMC6137411; doi:10.12688/f1000research.14819.2)
Supplement: Supplementary file 3 [file f1000research-7-17614-s0002.tgz › b2a79496-4ada-47c4-9682-a99ce867836d.zip!/Eight Iranian Health Journals/Archives of Rehabilitation_4.docx]

© Archives of Rehabilitation (RJ). University of Social Welfare and Rehabilitation Sciences, Koodakyar Alley, Daneshjoo Blv., Evin, Tehran, Iran. This work is licensed under [CC BY-NC 4.0](https://creativecommons.org/licenses/by-nc/4.0/).

Volume & Issue: دوره 18 - شماره 2

Title: رابطه ویژگی‌های شخصیتی با کیفیت زندگی همسران بیماران دچار ناتوانی جسمانی

http://rehabilitationj.uswr.ac.ir/article-1-1819-fa.html

هدف پژوهش حاضر با هدف تعیین رابطه ویژگی‌های شخصیتی با کیفیت زندگی در همسران بیماران دچار ناتوانی جسمانی انجام شد.  روش بررسی پژوهش حاضر، مطالعه‌ای پیمایشی از نوع همبستگی است که در سال 94-1393 انجام شد. جامعه این تحقیق را تمامی همسران بیماران دچار ناتوانی جسمانی تحت پوشش سازمان بهزیستی کرمان تشکیل می‌دهند. از این جامعه 100 نفر از همسران بیماران دچار ناتوانی جسمانی به روش در دسترس انتخاب شدند. برای سنجش متغیرهای پژوهش از پرسش‌نامه شخصیتی پنج‌عاملی نئو و پرسش‌نامه 36 سؤالی کیفیت زندگی واروشربون استفاده شد. داده‌ها با استفاده از نسخه 21 نرم افزار spss، آمار توصیفی، روش همبستگی و تحلیل رگرسیون تجزیه‌وتحلیل شد.  یافته‌ها نتایج حاصل از داده‌ها نشان داد بین ویژگی‌های شخصیتی مانند برون‌گرایی، دلپذیربودن و مسئولیت‌پذیری، با کیفیت زندگی همسران افراد دچار ناتوانی جسمانی رابطه مثبت و معنی‌داری وجود دارد (01/0P<). همچنین بین ویژگی شخصیتی روان‌نژندی با کیفیت زندگی همسران افراد دچار ناتوانی جسمانی رابطه منفی و معنی‌داری وجود دارد (01/0P<). نتایج حاصل از رگرسیون نشان داد که ویژگی شخصیتی روان‌نژندی به میزان 6/17 درصد و ویژگی شخصیتی برون‌گرایی به میزان 2/5 درصد واریانس کیفیت زندگی را در همسران افراد دچار ناتوانی جسمانی پیش‌بینی می‌کند (01/0P<).  نتیجه‌گیری با توجه به نتایج می‌توان استنباط کرد که ویژگی‌های شخصیتی مراقبان افراد معلول، ارتباط مؤثری با کیفیت زندگی آنان و به تبع آن بیماران دارد و بستر مناسبی را برای کاهش مشکلات ناشی از بیماری برای خانواده و بیماران فراهم می‌کند.

Title: بررسی ویژگی‌های روان‌سنجی برنامه تشخیص لکنت تمارضی

http://rehabilitationj.uswr.ac.ir/article-1-2048-fa.html

هدف لکنت نوعی اختلال در روانی گفتار محسوب می‌شود که از طریق وقفه‌های غیرارادی در جریان گفتار پیوسته مشخص می‌شود. لکنت انواع مختلفی دارد که عبارتند از: لکنت رشدی، اکتسابی و تمارضی. به دلیل وجود قانون معافیت از خدمت سربازی در مبتلایان به لکنت، مسئله تمارض در این حیطه موضوع بسیار مهمی است. در فرایند معافیت از خدمت سربازی ابزار استانداردی وجود ندارد و متخصصان بیشتر مبتنی بر قضاوت‌های ادراکی و غیررسمی و تشخیص شخصی به این امر مبادرت می‌ورزند. در سنجش‌هایی که قضاوت‌ها بیشتر از طریق راه‌های ادراکی و غیررسمی انجام می‌شود، احتمال خطا افزایش می‌یابد. هدف این مطالعه، ارائه یک برنامه علمی و بررسی ویژگی‌های روان‌سنجی آن به منظور تشخیص لکنت تمارضی از لکنت واقعی با هدف تعیین شرایط معافیت از خدمت سربازی است. روش بررسی این مطالعه از نوع مطالعات اعتباریابی بود که با روش نمونه‌گیری غیراحتمالی و از نوع ساده و در دسترس انجام شد. ابتدا برنامه تشخیص لکنت تمارضی تهیه شد که شامل بخش‌های مصاحبه ساختارمند، پرسش‌نامه‌ تشخیص لکنت تمارضی، پرسش‌نامه‌ شخصیتی چندمحوری مینه‌سوتا، تکنیک‌های تسهیل‌گر، آزمون ابزار سنجش شدت لکنت و تجزیه‌وتحلیل نمونه خواندن و گفتار توصیفی بود. به منظور بررسی ویژگی‌های روان‌سنجی برنامه، از 20 آزمودنی متقاضی معافیت از خدمت به دلیل لکنت که احتمال تمارض داشتند و 15 آزمودنی دارای لکنت رشدی با شدت لکنت متوسط تا شدید مراجعه‌کننده به کلینیک گفتاردرمانی که احتمال تمارض نداشتند، نمونه‌گیری انجام گرفت. داده‌ها با روش‌های مختلف آماری و به کمک نرم‌افزار SPSS تجزیه‌وتحلیل و بررسی شدند. یافته‌ها ضریب آلفای کرونباخ برای پرسش‌نامه‌ تشخیص لکنت تمارضی 76/0 به دست آمد. تعیین روایی هم‌زمان برنامه (نتایج حاصل از اجرای برنامه با نظر روان‌پزشکان) با استفاده از آزمون کای اسکور انجام شد. میان نظرهای دو گروه اختلاف معناداری یافت نشد (05/0P>).با استفاده از روش کندال همبستگی بین مؤلفه‌های مختلف متوسط و بالا برآورد شد. درنهایت برای هر مراجع براساس نتایج به‌دست‌آمده نیم‌رخ ترسیم شد. نتیجه‌گیری با توجه به نتایج به‌دست‌آمده می‌توان اظهار کرد برنامه تشخیص لکنت تمارضی همسانی درونی و روایی هم‌زمان مناسبی دارد، اما با توجه به اینکه حجم نمونه در این مطالعه بالا نبود، می‌توان این مطالعه را یک بررسی مقدماتی برای یافتن ویژگی‌های روان‌سنجی این ابزار دانست که با هدف بنانهادن پایه برای پژوهش‌های بیشتر در این زمینه اجرا شد.

Title: بررسی تأثیر ارتزهای مچ پا-پایی بر متغیرهای راه‌رفتن و تعادل افراد سکته مغزی: مطالعه مروری

http://rehabilitationj.uswr.ac.ir/article-1-1980-fa.html

هدف سکته مغزی به دنبال قطع یا اختلال خون‌رسانی به قسمتی از مغز ایجاد می‌شود. عوارض ناشی از سکته مغزی، می‌تواند از عوارض جزئی همانند علائم عصبی تا اختلالات خیلی شدید، بسته به محل و شدت آسیب‌دیدگی متفاوت باشد. همی‌پارزی یکی از شایع‌ترین اختلالات در افراد دچار سکته مغزی در فاز حاد این عارضه است. برخی اختلالات دیگر نیز همچون اختلالات تعادلی وضعیتی نیز ممکن است به دنبال سکته مغزی مشاهده شود. تمامی اختلالات نورولوژیکی ناشی از سکته مغزی ممکن است سبب محدودیت دامنه حرکتی افراد از رنج کم تا زیاد شود. بازیابی توانایی راه‌رفتن یکی از مهم‌ترین اهداف توان‌بخشی افراد دچار سکته مغزی است. از این رو استفاده از وسایل کمکی همچون ارتزها می‌تواند برای این افراد مفید واقع شود. یکی از متداول‌ترین روش‌های درمانی برای کنترل عارضه دراپ‌فوت ناشی از سکته مغزی استفاده از ارتزهای مچ پا-پایی است. ارتز مچ پا-پایی با فراهم کردن ثبات مچ و قرار دادن پا در وضعیت مطلوب از افتادگی پا و سایر حرکات ناخواسته جلوگیری می‌کند و باعث می‌شود تا راه‌رفتن عادی شود. هدف مطالعه مروری حاضر ارزیابی تأثیرگذاری ارتزهای مچ پا-پایی بر تعادل و مؤلفه‌های زمانی‌مکانی، کینتیکی و کینماتیکی راه‌رفتن افراد با عارضه دراپ‌فوت ناشی از سکته مغزی است. روش بررسی جست‌وجو در پایگاه‌های اطلاعاتی گوگل، اشپرینگر، ساینس‌دایرکت، پابمد، و اوید برای یافتن مطالعات در بازه زمانی 1996 تا 2016 که تاثیر ارتوزهای مچ پا- پایی را در افتادگی مچ سنجیده‌اند، انجام شده است. پس از بررسی و طبقه‌بندی مقالات به دست آمده، مقالات موجود بر اساس متغیرهای زمانی مکانی راه رفتن، کینتیک، کینماتیک و تعادل تحلیل شدند. در نهایت 21 مطالعه جهت ارزیابی نهایی انتخاب شدند. یافته‌ها 21 مقاله درباره تأثیر ارتز مچ پا-پایی بر متغیرهای راه‌رفتن افراد دراپ‌فوت ناشی از سکته مغزی تجزیه‌وتحلیل شده است. متغیر زمانی‌مکانی در 14 مقاله و مؤلفه‌های کینتیک و کینماتیک در هفت مقاله ارزیابی شده است. ارتز مچ پا-پایی تأثیر معناداری بر طول و عرض گام داشته است، ولی بر سرعت، کادنس، قرینگی راه‌رفتن و تعادل افراد تأثیر معنادار نداشته است. همچنین استفاده از ارتز مچ-‌پایی سبب بهبود مؤلفه‌های کینتیکی راه‌رفتن، کینماتیک مچ پا و کینماتیک زانو در فاز ایستایی می‌شود، ولی بر کینماتیک مفصل زانو در فاز نوسانی و کینماتیک مفصل هیپ در صفحه فرونتال و ساجیتال تأثیر چندانی نداشته است. درباره تأثیر ارتز بر ممان اینرسی و پاور مفاصل مطالعات اندکی وجود داشت، ولی نتیجه حاکی از نبود تفاوت معنادار در این مؤلفه‌ها بود. نتیجه‌گیری این مطالعه نشان داد مدل های مختلف ارتز مچ پا-پاییاستاتیک یا دینامیک، بسته به جنس و طراحی‌شان، تأثیرهای متناقضی بر تعادل، مؤلفه‌های کینتیک، کینماتیکی و متغیرهای راه‌رفتن افراد سکته مغزی می‌گذارند. استفاده از ارتزهای مچ پا-پایی در مقایسه
